# Breast bud detection: a validation study in the Chilean Growth Obesity Cohort Study

**DOI:** 10.1186/1472-6874-14-96

**Published:** 2014-08-13

**Authors:** Ana Pereira, María Luisa Garmendia, Daniela González, Juliana Kain, Verónica Mericq, Ricardo Uauy, Camila Corvalán

**Affiliations:** 1 Institute of Nutrition and Food Technology, University of Chile, Avda. El Líbano 5524, Macul, Santiago, Chile; 2 Institute of Maternal and Child Research, Faculty of Medicine, University of Chile, Avenida Santa Rosa N° 1234, 2° piso, Santiago, Chile; 3 Department of Pediatrics, Faculty of Medicine, Catholic University of Chile, Santiago, Chile

**Keywords:** Puberty onset, Sexual development, Puberty cohorts

## Abstract

**Background:**

Early puberty onset has been related to future chronic disease; however breast bud assessment in large scale population studies is difficult because it requires trained personnel. Thus our aim is to assess the validity of self and maternal breast bud detection, considering girl’s body mass index (BMI) and maternal education.

**Methods:**

In 2010, 481 girls (mean age = 7.8) from the Growth and Obesity Chilean Cohort Study were evaluated by a nutritionist trained in breast bud detection. In addition, the girl(n = 481) and her mother(n = 341) classified the girl’s breast development after viewing photographs of Tanner stages. Concordance between diagnostics was estimated (kappa, Spearman correlation) considering girls’ BMI and mother’s educational level.

**Results:**

14% of the girls presented breast buds and 43% had excess weight (BMI z-score > 1, World Health Organization 2007). Self-assessment showed low concordance with the evaluator (K < 0.1) and girls with excess weight over-diagnosed more than girls of normal weight (44% vs. 24%, p-value < 0.05). Instead, mothers showed good concordance with the evaluator (K = 0.7, 95% confidence interval (CI) = 0.6-0.9), even in overweight girls and/or in mothers with low education (K = 0.7, 95% CI = 0.6-0.8).

**Conclusions:**

Mothers were able to adequately evaluate the appearance of breast bud despite low educational level and girls’ excess weight. Mother could be a useful resource for defining puberty onset in epidemiological studies, particularly developing countries.

## Background

Traditionally, puberty has been defined by age at menarche. However, there is widespread agreement that the period of time from onset of puberty to menarche varies greatly and it had lengthened in the last decades [1]. For epidemiological studies, it is important to describe the age of onset as well as how quickly it progresses, because the former has been related to future chronic diseases at adulthood [2,3].

Appearance of breast bud is the first indicator of puberty onset in 90% of the girls. Clinical and epidemiological studies commonly use visual inspection to assess the appearance of breast bud based on photographs (Tanner Breast Stage 2 (B2)) proposed by Marshall and Tanner in the 1960s [4]. However, this assessment is sometimes difficult to carry out in epidemiological studies because of the difficulty in providing training to evaluators and also the need to perform evaluations in places with adequate privacy; cultural and religious barriers may also exist.

Tanner self-assessment has been proposed as an alternative in various studies [5]. This involves showing photographs (with or without explanatory text) to the girl allowing her to examine at a mirror to categorize her stage of breast development [6]. However, the validity of this evaluation vary (Kappa statistic (K) compared to trained personnel =0.4-0.8) (4, 5), and differences by race and body mass index have been described [7].

Another alternative is that the child’s mother evaluates her daughter’s breast staging. We are not aware of studies assessing the validity of this evaluation but a high correlation with the medical diagnosis (r = 0.8) has been described [8].

However, a major restriction posed by all methods based on visual inspection is that fat tissue is difficult to differentiate from breast bud [9]. This is an increasing problem given the current obesity epidemic affecting children and adolescents worldwide. Thus, breast palpation is considered the gold standard technique for detecting breast bud [10].

The Growth and Obesity Chilean Cohort Study (GOCS) aims to evaluate the relationship between early life nutrition and later growth and development in a representative sample of Chilean girls from middle- and low-income families. As part of this study, trained personnel, the girls (self-assessment) and the girls’ mothers evaluate the appearance of breast buds. The purpose of the present study is to compare the agreement between self- and maternal B2 assessment in relation to trained personnel during the evaluations carried out in 2010 considering potential differences by nutritional status and the mother’s educational level.

## Methods

### Study design

During 2010 we carried out a cross-sectional validation study within the GOCS cohort study. Original GOCS participants were 1,196 children (601 girls) of low- and middle-income social status selected from preschools registered with the National Board of Preschool Council Program (*Junta Nacional de Jardines Infantiles*) in southeastern Santiago. The sample included children born in 2002 and 2003 from a single, full-term pregnancy (≥37 weeks) with a birth weight ranged 2,500 - 4,500 grs who had no evidence of disease at birth. Chilean population is predominantly of mixed Spanish-indigenous ethnic origin, with < 5% native indigenous people [11]. As part of the study GOCS participants are invited annually at the Institute of Food Nutrition and Technology of the *Universidad de Chile* in Santiago Chile to carry out detailed evaluations including Tanner assessments.

### Study population

**Figure 1:**
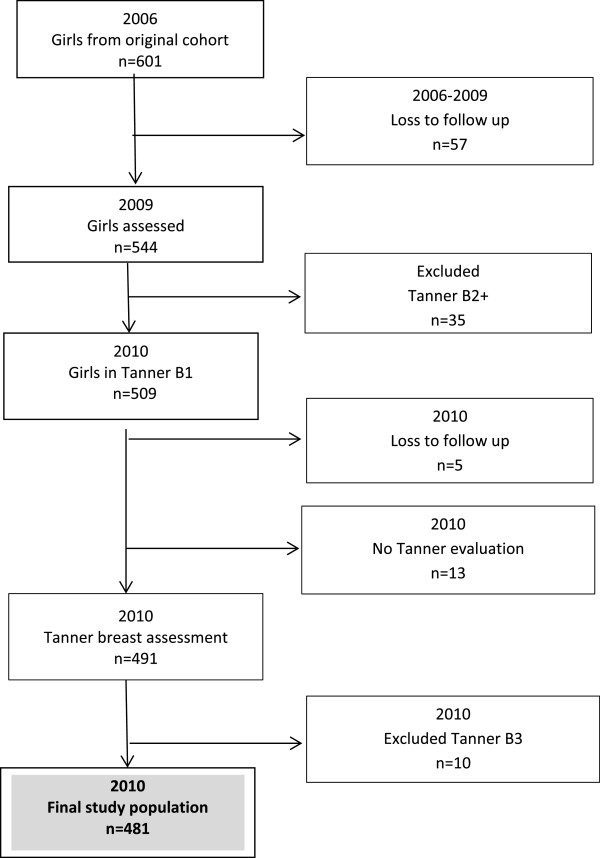
Study population included in the study, Santiago, 2010.

From 2006 to 2009, we lost 57 girls from the original cohort (tracking loss at 4 years: ~10%):15 changed their place of residence, 11 could not be located, 29 declined to participate in the study and 2 were excluded due to medical disorders (Figure 1); thus, in 2009 we evaluated 544 girls of whom 509, did not have developed breast bud. In 2010, we lost 5 girls (3 because changed residence and 2 were not located) 13 girls did not accepted Tanner breast evaluation, and 10 girls were evaluated as B3, thus our final sample size was 481 of whom 340 were also evaluated by the mother. Assuming a Kappa of 0.7-0.8 and B2 prevalence of 25% [12] a sample size of 340 will have an absolute precision of 5 to 6%. No significant differences were found between girls contacted and those lost to follow up in relation to mother’s education, birth weight and length and nutritional status at five years old (p > 0.05).

### Measurements

The evaluation of breast bud was conducted by a nutritionist trained specially for this purpose; the evaluation included inspection and palpation of the breasts [4] using standard methods (K value for B2 comparison of nutritionist and pediatric endocrinologist = 0.9, n = 100). Appearance of the breast bud was defined as breast tissue under the areola but not beyond it, protrusion of the areola and nipple and increased diameter of the areola (1, 13). Once the nutritionist’s evaluation was completed, both the girl and her mother were shown standard Tanner staging photos (in black and white) [4] and were requested to classify breast development without any further explanation of how to interpret them. Mothers and daughters breast bud evaluation was only based on visual inspection. The breast bud assessment of the three evaluators (trained nutritionist, mother and daughters) were done independently and blinded from each other’s.

A trained nutritionist carried out the anthropometric measurements (weight and height) of all the girls at the visit in light cloths and barefoot. Weight was measured with a Tanita 418 BC (accuracy within 0.1 kg) and height was measured with a Seca 222 measuring rod (accuracy within 1 mm). Body mass index (BMI) was calculated as weight/height^2^ (kg/m^2^) and the z-score was calculated based on the World Health Organization (WHO) 2007 curves. The girls’ nutritional status was classified as follows: underweight (BMI z-score < -1 ), normal (BMI z-score -1 to 1), overweight (BMI z-score >1 to 2), obesity (BMI z-score > 2) [13]. The mother of the girls answered a questionnaire in relation to the years of formal education received and it was dichotomized in less than eight versus eight or more years of formal education (in accordance with definition of primary schooling level in Chile).

### Statistical analysis

Breast tanner staging was dichotomized as B1 or B2. The concordance or agreement (K and 95% confidence intervals (CI)) among the various evaluations (self-assessment, maternal and trained personal evaluations) were estimated. Concordance was also calculated according to the girl’s nutritional status (BMI z-score ≤ 1 (underweight and normal weight) vs BMI z-score > 1 (overweight/obesity)) and maternal education (<8 yrs of study vs ≥ 8 yrs of study). A K of 0.81-1.00 was considered "Very good" agreement, "good" was defined as 0.61-0.80, "moderate" as 0.41-0.60, "weak" as 0.21-0.4 and "poor" as < 0.20 [14]. This analysis was also repeated calculating Spearman correlations.

Sensitivity (percentage of girls or mothers who correctly diagnosed the presence of breast buds) and specificity (percentage of girls or mothers who correctly diagnosed Tanner breast stage 1) of breast bud detection was calculated using the trained personnel assessment as the reference. Over-diagnosis of breast bud was estimated as the percentage of the girls classified as Tanner stage 1 by the trained personnel who were evaluated as Tanner stage 2 by the girl or her mother. These analyses were repeated, stratifying by the girl’s nutritional status and mother’s education; statistical differences among groups were evaluated using Chi-squared test and were considered significant if p-value <0.05. All analyses were performed with STATA version 10.0 [15].

### Ethical aspects

GOCS study protocol and consent forms were approved by the Ethics Committee of the Institute of Nutrition and Food Technology of the *Universidad de Chile*. Mothers signed an informed consent form and each girl also signed a form indicating her agreement to participate in the study. Tanner evaluations were performed by a single female nutritionist in the presence of a responsible adult (in case they did not attended the visit with the mother) and in conditions of absolute privacy.

## Results

The 481 girls had on average 7.8 years (6.8 to 9.2 yrs) at the time of evaluation; and 14% were classified as breast Tanner stage 2. Almost half of the girls had excess weight (26% overweight and 16% obese) and most attended with their mothers (71%) of whom 35% had less than eight years of education (Table 1).

**Table 1:** Characteristics of the study population, stratified by number of evaluation, Santiago 2010

	**N**	**%**
Total	481	
Age (yrs) (mean, SD*)	7.83	0.4
Body Mass Index (kg/mt2)		
Underweight (< -1DS)	19	4.0
Normal Weight (-1 DS- 1DS)	257	53.4
Overweight (1-2DS)	127	26.4
Obesity (>2DS)	78	16.2
Mother’s Schooling (yrs)		
< 8 years	167	35.1
≥ 8 years	309	64.9
Attending with mother		
Yes	341	70.9
No	140	29.1
Breast Tanner stage at moment of assessment		
B1	412	85.7
B2	69	14.4

*SD standard deviation.

The agreement between self-assessment of breast bud and the evaluation conducted by the mother in relation to trained personnel is shown Tables 2 and 3. Concordance between self-assessment and trained personnel was poor (K = 0.02; 95% CI:- 0.06; 0.10. Spearman correlation: 0.03). Only 36% of the girls, who were classified as Tanner stage 2 by trained personnel, found they had breast buds (sensitivity), while 68% of girls in Tanner stage 1 correctly assessed their stage (specificity). After stratifying by nutritional status, self-assessment continued to show low agreement and correlation with the evaluation by the trained personnel (Table 2); however the over diagnosis was significantly higher in girls with excess weight (43.6% vs 24.3%, p-value < 0.05).

**Table 2:** Agreement between breast buds diagnosed by the girls and trained personnel, stratified by girl’s BMI z-score

	**Girl’s Breast Tanner rating**	**Breast Tanner rating by trained personnel**			
		**Tanner 2 +**	**Tanner 1**	**Kappa index**	**Spearman**
		**N (%)**	**N (%)**	**(95% CI)**	
	***Study population***				
Entire Study Population (n = 474)	Tanner 2	24 (35.8)	132 (32.4)	0.02 (-0.06; 0.10)	0.03
	Tanner 1	43 (64.2)	275 (67.6)		
	Total	67	407		
	***Stratified by girls’ BMI z-score at assessment***				
Girls’ BMI z-score ≤ 1DS (n = 274)	Tanner 2	14 (35.9)	57 (24.3)	0.09 (0.004; 0.17)	0.09
	Tanner 1	25 (64.1)	178 (75.7)		
	Total	39	235		
Girls’ BMI z-score > 1DS (n = 200)	Tanner 2	10 (35.7)	75 (43.6)	-0.04 (-0.12; 0.03)	-0.06
	Tanner 1	18 (64.3)	97 (56.4)		
	Total	28	172		

*BMI: Body Mass Index.

**Table 3:** Agreement between breast buds diagnosed by the girls’ mother and trained personnel, stratified by girl’s BMI z-score and mother’s schooling

	**Mother’s Breast Tanner rating**	**Breast Tanner rating by trained personnel**			
		**Tanner 2 +**	**Tanner 1**	**Kappa index**	**Spearman**
		**N (%)**	**N (%)**	**(95% CI)**	
	***Study population***				
Entire Study Population (n = 341)	Tanner 2	49 (89.1)	22 (7.7)	0.73 (0.63; 0.82)	0.74
	Tanner 1	6 (10.9)	264 (92.3)		
	Total	55	286		
	***Stratified by girls’ BMI z-score at assessment***				
Girls’ BMI z-score ≤ 1DS (n = 204)	Tanner 2	27 (87.1)	11 (6.4)	0.74 (0.65; 0.83)	0.74
	Tanner 1	4 (12.9)	162 (93.6)		
	Total	31	173		
Girls’ BMI z-score > 1DS (n = 137)	Tanner 2	22 (91.7)	11 (9.7)	0.71 (0.62; 0.81)	0.73
	Tanner 1	2 (8.3)	102 (90.3)		
	Total	24	113		
	***Stratified by mother’s schooling***				
Mother’s schooling < 8 years (n = 123)	Tanner 2	23 (88.5)	11 (11.3)	0.69 (0.59; 0.79)	0.70
	Tanner 1	3(11.5)	86 (88.7)		
	Total	26	97		
Mother’s schooling ≥ 8 years (n = 216)	Tanner 2	26 (89.7)	10(5.4)	0.77 (0.68; 0.85)	0.77
	Tanner 1	3 (10.3)	177 (94.7)		
	Total	29	187		

*BMI: Body Mass Index.

The agreement between the breast bud evaluation by the mother and that of the evaluator was good (K = 0.7, 95% CI: 0.6; 0.8 spearman correlation of 0.7) with a sensitivity and specificity of 89% and 92%, respectively. No significant differences were observed by the girl’s nutritional status; however mothers of leaner girls had one third less probability to over-diagnose breast buds. Mothers with > 8 yrs of education showed greater inter-observer agreement and lower rates of over-diagnosis of Tanner stage 2 than the less-educated mothers (5.4% vs. 11.3% respectively); though these differences were not statistically significant (p-value >0.05). Of note, even the evaluations by less educated mothers showed good concordance with those conducted by the trained examiner (K = 0.7) (Table 3).

## Discussion

The findings of our study demonstrate that Chilean mothers of low- and middle-income status can be good evaluators of breast bud appearance in their daughters, even if their daughters present excess weight. The evaluation performed by the girl herself was not reliable, one out of two girls over-diagnosed breast bud.

To our knowledge, only one previous study has evaluated the reliability of the girl’s mother as an evaluator of pubertal development using Tanner scale. Using a sample of 151 high-income girls in the United States, Brooks-Gunn et al., showed that mothers (85% had higher education studies) had a better correlation for assessing breast development than their daughters (r = 0.8 vs 0.7 respectively) [8]. Although the report did not include concordance findings, based on the tables published we estimated that the Kappa statistic for determining breast development in Tanner stage 2 by the mother was only 0.40. These mothers were not specifically trained in Tanner evaluation, but rather received instructions by mail and carried out evaluations in their homes.

Several studies have evaluated the validity of self-assessment of mammary buds, but results vary greatly. Some reports found concordance greater than 0.7 between self-examination and trained personnel [6,16-18], while others found values less than 0.5 [10,19-21]. Our results on self-assessment agreement by girls are far below those reported by other authors. This may be due to the fact that our reference method was subject to less error as we used breast palpation and we carried out evaluation in clinical settings as recommended in the literature [22]. Also, our method for self-assessment was based on the use of black and white photographs without additional verbal or written explanations or opportunity for the girls to look themselves in a mirror. There is evidence that concordance increases when real photographs in color are used, when supporting texts with a short description of each image are used and adapted to local language [16], or if the girl has the opportunity to look at herself in a full-length mirror during the self-assessment [6]. Finally, another possible reason for the low agreement of the self-assessment is the young ages of our children which ranged from 6–9 years old [23,24].

It is also possible that our results reflect cultural differences with respect to sexual care and behavior. Chile is a country with a conservative sexual behavior policy. Sexual anatomy or education is not part of the school curricula and contraceptives are not provided freely to minors. As a result, national surveys report a low level of sexual education of adolescents [25]. This in addition to the young age of the daughters, we may believe that our girls have a very low level of knowledge in relation to sexual education and anatomy, which will lead to find a low concordance in the self-assessment of breast bud in this group. Conversely, parents are expected to play a role in children care well advanced adolescence. For example, national guidelines on infant care recommend that between 8–12 years children should be gradually responsible for their personal care [26], thus mothers are involved in bathing their children until they are approximately 8-10y of age; which could explain their high concordance in breast bud evaluation.

Obesity significantly increases the difficulty of detecting the appearance of breast buds. Obese girls have worse concordance with trained personnel and tend to over-diagnose their mammary development due to the presence of lipomastia [23,27]. A study of 9,132 Chinese girls found that the Kappa statistic for evaluating Tanner breast stage 2 was 0.62 among non-obese girls, while it was only 0.53 among obese girls; moreover, almost one in five girls in the latter group over-diagnosed their mammary development [27]. In our study we found that maternal report had a good agreement with trained report even in obese girls; however, as suggested, we found greater overestimation of mammary development in girls with overweight/obesity (44% vs. 24%). We found that maternal education used as a proxy for socioeconomic status was not a restriction for mother’s capacity to conduct pubertal evaluation of their daughter. This finding is of great practical importance for large-scale epidemiological studies particularly in the context of developing countries in which education may still be a limitation.

Some limitations should be taken into account when interpreting our results. Given the young age of the girls in our sample, only a small percentage of them presented breast bud appearance (20%), these girls had more excess weight (49% vs. 43%) and were taller (>2SD, 9.2% vs. 1.8%) than girls who did not yet present breast bud appearance. However, the direction and magnitude of our results, in terms of the mother’s assessment, are so clear that we believe that although numbers may vary, the main conclusions of our study will remain as more girls in the cohort mature. Also, we cannot exclude that the low concordance observed with self-reported breast bud appearance in our study is due to the young age of our participants. Moreover, our results only apply to breast bud appearance (Breast Tanner 2) and cannot be extrapolated to further Tanner stages. Studies carried out at older ages provide mixed results [7,16,27]; thus, it would be interesting to see how our cohort behaves at later ages. On the other hand, our study has the strength that it involved examiners trained in the palpation method for breast buds, providing a true "gold standard" against which to compare the observations of the mothers and the girls. Also, given that Tanner evaluations were part of a larger study, we were also able to quantify the potential effect of adiposity and the observer’s educational level in the validity of these evaluations.

## Conclusion

In conclusion, our results suggest that epidemiological studies aimed at describing and evaluating the determinants of pubertal events can use mothers to diagnose the breast bud appearance of girls, even when the girls have excess weight or the mothers have a low educational level. Validation of simpler methods of diagnosis of puberty onset is promising, as its use can be extended to population studies leading to a better understanding of one of the early windows of susceptibility to adult health.

## Abbreviations

BMI: Body mass index; CI: Confidence interval; GOCS: Growth and Obesity Chilean Study; K: Kappa index; SD: Standard deviation; WHO: World Health Organization.

## Competing interests

The authors declare that they have no competing interests.

## Authors’ contributions

AP: participated in study design, carried out analysis, and drafted the manuscript. MLG: carried out analysis, review and revised in detail the manuscript and participated in drafting the initial manuscript. CC: conceptualized and designed the study, and critically reviewed the manuscript. DG: coordinated data collection and cleaning dataset and participated in elaborating initial manuscript. JK: participated in the design of the study and revised the manuscript and approved the final manuscript as submitted. VM: participated in interpreting and analysis of data and revised the manuscript. RU: contributed to the design of the study and in the interpretation of data. All authors read and approved the final manuscript as submitted.

## Pre-publication history

The pre-publication history for this paper can be accessed here:

http://www.biomedcentral.com/1472-6874/14/96/prepub
